# From “classic” child abuse and neglect to the new era of maltreatment

**DOI:** 10.1186/s13052-017-0336-1

**Published:** 2017-01-28

**Authors:** Pietro Ferrara, Sergio Bernasconi

**Affiliations:** 1 Institute of Pediatrics, Catholic University Medical School, Rome, Italy; 2 Service of Pediatrics, Campus Bio-Medico University, Rome, Italy; 3 Pediatrics Honorary Member University Faculty, G. D’Annunzio University of Chieti-Pescara, Chieti, Italy

**Keywords:** Child abuse, Neglect, Maltreatment

## Abstract

The evolution of the concept of child abuse leads to consider new types of maltreatment that in the future will certainly be taken into account with a new era of social pediatrics.

Pediatric care has been based on the increased awareness of the importance of meeting the psychosocial and developmental needs of children and of the role of families in promoting the health.

According to federal definition of Child Abuse Prevention and Treatment Act (CAPTA) by the term “child abuse and neglect” we mean any act, or failure to act, by a parent or caregiver, meaning by this term the teacher, coach and anyone who has an educational role or caregiving, which leads to physical or emotional harm, sexual exploitation or abuse, or death; or an act, or failure to act, that results in imminent risk of injury [1]. The negative experience of each child affects greatly his or her health from a physical, mental and social point of view with huge socio-economic costs for each type of abuse [2].

The evolution of the concept of child abuse leads to consider new types of maltreatment that in the future will certainly be taken into account with a new era of social pediatrics. All adverse childhood experiences (ACEs), typically defined as stressful or traumatic life events that occur during the first years of life, such as emotional, physical, or sexual abuse, emotional or physical neglect, or other forms of family dysfunction, are pervasive and notable public health problems.

First of all the number of refugees who attempt to reach Italy is increasing and many of these children are alone. These children may fall victim of kidnapping, trafficking, labour and sexual exploitation, prostitution. Then number of female children who disappear yearly is higher that the male one: up to 50% of female minors vanish yearly, while only 30% of them disappeared during the last year [3, 4]. However, the phenomenon of the unaccompanied migrant minors also has expanded to different parts of Europe, as new migratory routes to the north of Europe have recently emerged. All separated children have the right to be clothed, fed, and accommodated, and to receive proper health care, to be educated, and to be informed in a language they understand to contain the negative outcomes of this phenomenon.

Another interesting new aspect is the long stay of children in foster care (FC) and the consequences on mental and physical health status. Foster care is a social system based on institutions, group homes or private homes for orphaned, abandoned and maltreated children [5, 6]. FC’s aim is to provide a temporary refuge for children at risk for damage, safeguarding their well-being, and to assist birth parents in their needs, to reunite them with their children. Removing children from their primitive family and admitting them to foster care can be a hard choice: many stressors can with the placement. Furthermore, children in FC are physiologically and emotionally subjected to constant remodeling to their environment: toddlers, in child wellbeing, often have a stress response that is deregulated. Even if mental health services are provided for foster children, sometimes they could be inadequate to their needs. In fact, they should be able to identify risk factors, anticipating the detection of child abuse and improving the quality of life that FC supply, leading to a forward mental health disorder’s diagnosis. The hosted children appear to be, in neuropsychiatric and neurological terms, very fragile and needy of constant care and periodical specialistic revaluation.

Further actual impact on social pediatrics is represented by the new types of families: these changes can be attributed to several factors, including high divorce rates and heterogeneous family structures, that extend beyond biological or conjugal relationship boundaries. These factors have been investigated over the years for possible psychological and physical risk factors to child health, which require further attention [7, 8]. Children’s psychosocial development is linked both to their relationships with parents and the sociocultural context in which they live. Pediatricians should be trained to play a major role in caring for and supporting the social and developmental well-being of children raised in variously configured families. With regard to the results of the studies and their limitations reported above, and the debates still open within the civil society of adults on children growing up in diverse families, it is important to emphasize that children’s well-being relies primarily on the parents’ competence and sense of security, as well as the presence of social and economic support for the family. Psychological and physical health are not so much based on the gender or the sexual orientation of their parents.

More attention should be given to children whose mother was murdered. The intimate partner homicide often involve the murder of family members or bystanders, such as the couple’s children, relatives or new partners of the victims and have long-term consequences on remaining family members [9, 10]. Little is known about the number of orphaned children who have instantaneously lost both parents. Children whose father kills their mother are orphaned by this act: they not only lose their mother, but also their father to prison or suicide. Placement after the death is very problematic. The caregivers often exert pressure on the child to forget what happened by not speaking about it, negating the child’s version of events, and responding with silence and evasion to the child’s questions so that the children will have difficulty to effectively mourn their losses. long-term studies are needed to ascertain what happens to these children (especially when they grow up), to understand what are the most appropriate psychological treatments, the best decisions about the contact with their father (when he is the murderer) and the best placement for these children.

Another emerging issue is represented by the injuries in stationary vehicles for children younger than 14 years old are poorly recognized type of vehicle injury and receive far less attention than motor vehicle crashes [11, 12]. Although this information is important, a lot of children die from heat stress because they have been left in closed automobiles, but few data regarding the circumstances surrounding the fatal event are available. Previous researches confirm that the leading cause of death for children is stroke and hyperthermia after being left unattended in motor vehicles. A recent explanation could be related to the Working Memory (WM). WM refers to the system or systems that are assumed to be necessary in order to keep things in mind, while performing complex tasks such as reasoning, comprehension and learning [13, 14]. There is great evidence that stress and enhanced glucocorticoids levels can influence memory performance with both negative and positive consequences. A recent study assessed the effects of stress and cortisol on a variety of memory tasks in male human subjects and demonstrated that there is a stress-induced WM impairment. Leaving a child alone in a car can be considered a form of child neglect.

Further attention has been paid to the health care of children under the age of 3 years in jail with their mothers [15]. Although there is poor direct evidence about the long-term effects of separating or keeping mothers with their babies while in prison, the role of the family as an essential element for mental and emotional development of the child is well known. For the first few years of life the interactive context of children coincides with the maternal figure and the mother’s psyche becomes an integral part of the child’s mind. More attention should be paid to healthcare promotion by providing healthcare educational programmes, with periodic meetings on important themes such as breast-feeding, infant sleeping position, accident prevention, first steps, alcohol and drugs use.

In conclusion, childhood maltreatment in any type manifests seems to be another risk factor for physical diseases, such as obesity, growth failure, lead poisoning, untreated vision problems, atopic dermatitis, infectious diseases [16]. In conclusion, as society and culture have progressively changed different configurations of child abuse and neglect have emerged. Pediatricians should be trained to play a major role in caring for and supporting the social and developmental well-being of children raised in variously conditions and in new types of problems [17–19]. Pediatric care has been based on the increased awareness of the importance of meeting the psychosocial and developmental needs of children and of the role of families in promoting the health.
